# In Vivo Evaluation of a Miniaturized Fluorescence Molecular Tomography (FMT) Endoscope for Breast Cancer Detection Using Targeted Nanoprobes

**DOI:** 10.3390/ijms21249389

**Published:** 2020-12-09

**Authors:** Hao Yang, Weipin Qian, Lily Yang, Huikai Xie, Huabei Jiang

**Affiliations:** 1 Department of Medical Engineering, University of South Florida, Tampa, FL 33612, USA; haoyang@usf.edu; 2 Department of Surgery, Emory University, Atlanta, GA 30322, USA; wqian@emory.edu (W.Q.); lyang02@emory.edu (L.Y.); 3 Department of Electrical and Computer Engineering, University of Florida, Gainesville, FL 32611, USA; xie@ieee.org

**Keywords:** breast cancer, patient-tissue-derived xenograft (PDX) tumor, nanoparticles, NIR dye, fluorescence molecular tomography, endoscope

## Abstract

In this study, in vivo animal experiments with 12 nude mice bearing breast-cancer-patient-tissue-derived xenograft (PDX) tumors were performed aiming to verify the imaging capability of a novel miniaturized fluorescence molecular tomography (FMT) endoscope, in combination with targeted nanoparticle–near-infrared (NIR) dye conjugates. Tumor-bearing mice were divided into two groups by systematic injection with urokinase plasminogen activator receptor-targeted (*n* = 7) and nontargeted (*n* = 5) imaging nanoprobes as a contrast agent, respectively. Each mouse was imaged at 6, 24, and 48 h following the injection of nanoprobes using the FMT endoscope. The results show that systemic delivery of targeted nanoprobes produced a 4-fold enhancement in fluorescence signals from tumors, compared with tumors that received nontargeted nanoprobes. This study indicates that our miniaturized FMT endoscope, coupled with the targeted nanoparticle–NIR dye conjugates as a contrast agent, has high sensitivity and specificity, and thus great potential to be used for image-guided detection and removal of a primary tumor and local metastatic tumors during surgery.

## 1. Introduction

Breast cancer is the most common cancer and the second leading cause of cancer death in women after lung cancer [1,2]. Triple-negative breast cancer (TNBC) accounts for about 20% of breast cancer patients, is usually detected at a late stage with aggressive pathological features, has an increased likelihood of local and distant recurrence, and has a short overall survival time [3,4]. Over 30% of TNBC patients develop a local recurrence, a distant metastasis, or both within the first three years of initial treatment, and most cancer deaths occur within five years of diagnosis [5,6]. Pre-operation neoadjuvant chemotherapy is usually given to TNBC patients to reduce the incidence of local and distant tumor recurrence. However, up to 50% of TNBC patients are highly resistant to chemotherapy, and the prognosis of those patients is very poor [6,7]. In order to increase the overall survival of TNBC patients, there is an urgent need to develop novel imaging technologies that can assist in the specific and sensitive detection of tumor lesions for surgical removal and to further reduce the incidence of local and distant tumor recurrence.

Over the past few decades, rapid development of several fluorescence imaging approaches has been prompted—benefiting from the increased use of nontargeted or targeted imaging agents in clinical applications and pre-clinical research. Fluorescence reflectance imaging (FRI), as one of the most common fluorescence imaging methods, is widely used for in vivo and ex vivo imaging in animals and humans, and is becoming an important tool for intraoperative tumor imaging and image-guided surgery [8,9,10]. While the FRI used in these studies is easy to set up and implement, its applicability is limited by its superficial imaging depth of only few millimeters and its incapability of quantitative imaging. As a convenient and cost-effective optical imaging modality, fluorescence molecular tomography (FMT) can overcome the limitations associated with FRI or other planar imaging methods and can provide accurate visualization and quantification of the three-dimensional (3D) distribution of fluorescent targets in deep turbid tissue. In FMT, the 3D spatial distribution of fluorescent probes is quantitatively recovered through a mathematical inverse algorithm using tomographically measured optical data along the boundary of the object [11,12,13,14]. Today, FMT is widely used in biomedical research and clinical studies, such as cancer imaging, stem cell trafficking, drug delivery, and enzyme activity monitoring [15,16,17]. The endoscope is a sophisticated optical instrument that is able to investigate the inside of the human body, especially the interior of a hollow organ or a cavity of the body. While great progress has been made in the last few decades [18,19,20], the design and development of an FMT-based endoscope remains a major challenge to the field due to the complex structure of FMT and the high requirements for miniaturization.

In this study, we propose a highly miniaturized FMT endoscope that uses a microelectromechanical systems (MEMS) mirror to perform 2D scanning and an optical fiberscope to capture multiple planar fluorescence images. This endoscopic device has a diameter of only 5 mm, the smallest FMT endoscope thus far, to our knowledge. Through extensive in vivo animal experiments in a breast cancer mouse model, we demonstrate the image capability of this miniaturized FMT endoscope and its potential as an intraoperative device for tumor removal. TNBC patient-tissue-derived xenograft (PDX) tumors were generated by subcutaneously implanting tumor fragments of an established PDX tumor from a surgical resected breast cancer tissue into the back flank of nude mice. Two animal groups were divided by systematically injecting one group with urokinase plasminogen activator receptor (uPAR)-targeted imaging nanoprobes and near-infrared 830 dye-labeled magnetic iron oxide nanoparticles (NIR 830-ATF-IONP) [21,22], and the other group with nontargeted near-infrared 830 dye-labeled magnetic iron oxide nanoparticles (NIR 830-IONP). The NIR 830 dye-labeled fluorescent probes were an optical imaging contrast agent. Compared with peptide imaging probes, targeted nanoparticles have a longer blood half-life, which increases the accumulation of the probes in tumors. Both FMT scanning and planar fluorescence imaging were performed at three different time points, and the imaging results obtained between different groups were compared and analyzed.

## 2. Results

Twelve nude mice (5–7 weeks old) were subcutaneously implanted with approximately 1 mm^3^ PDX tissue fragments (subcutaneous model). All nude mice had tumors grown successfully within weeks following implantation. The PDX tumors had similar morphological, pathological, and phenotypic characteristics as those of the primary tumor tissues from the patients. Once the tumor size was ready for imaging (4–6 mm after approximately 4 weeks of tumor implantation), targeted or control iron oxide nanoparticles (IONPs) in about 150 μL of saline solution were injected intravenously (i.v.) through the tail veins. For comparison, the 12 mice were divided into two groups by administering two different types of IONPs, respectively:

(1) Five mice: Nontargeted NIR 830-IONP
(2) Seven mice: Targeted NIR 830-ATF-IONP

Tumors were imaged at 6, 24, and 48 h following the injection using a miniaturized FMT endoscope. For comparison, planar fluorescence imaging was also performed subsequently at each time point. During the imaging, mice were anesthetized with isoflurane. Animals were placed on a warm pad or a heated blanket during the surgery and imaging procedures.

**Figure 1:**
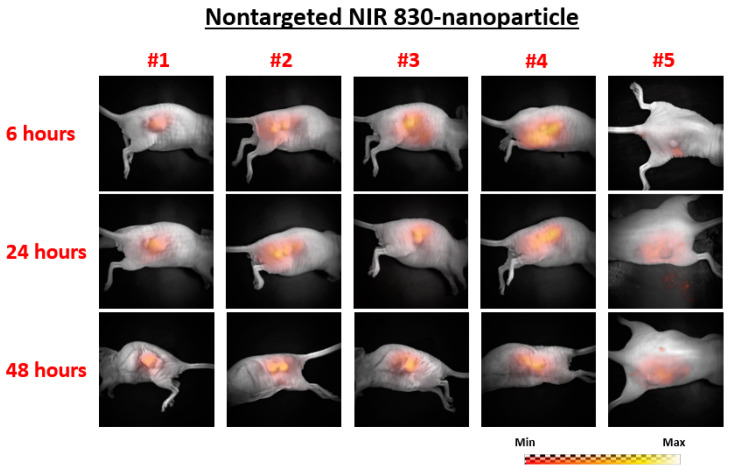
Planar fluorescence images of the five mice in the nontargeted nanoparticle–near-infrared 830 (nontargeted NIR 830) group at 6, 24, and 48 h post injection of nanoprobes.

**Figure 2:**
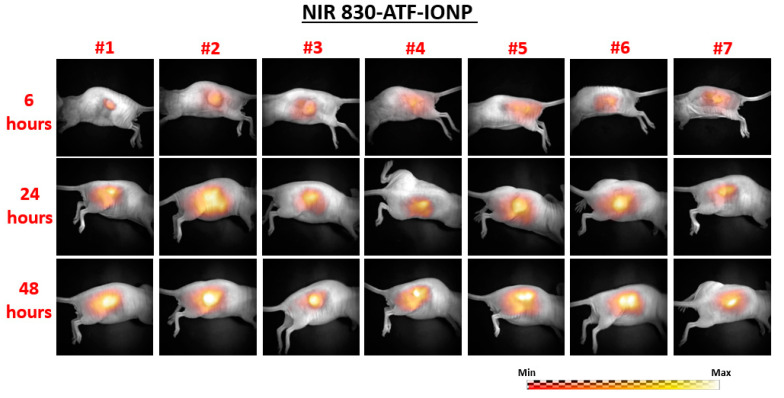
Planar fluorescence images of the seven mice in the urokinase plasminogen activator receptor (uPAR)-targeted imaging nanoprobes and near-infrared 830 dye-labeled magnetic iron oxide nanoparticles (NIR 830-ATF-IONP) group at 6, 24, and 48 h post injection of nanoprobes.

**Figure 3:**
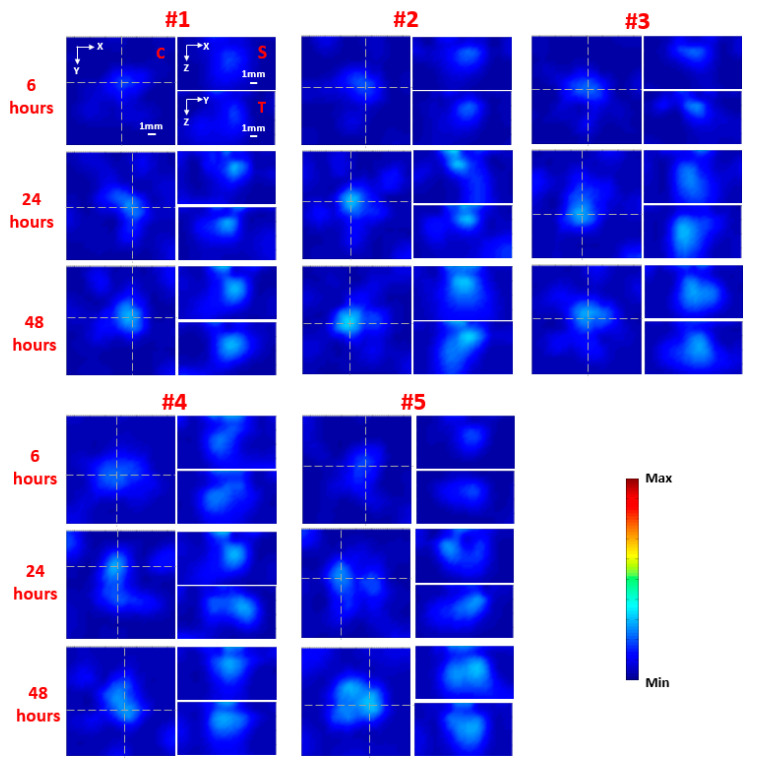
Reconstructed fluorescence molecular tomography (FMT) images along representative coronal (C), sagittal (S), and transverse (T) planes for the five mice from the nontargeted (nontargeted NIR 830) group at 6, 24, and 48 h post injection of nanoprobes. The C slice is indicated in the first image for mouse #1 (upper left), while the S and T slices are also noted in the other two images in the first set of images for mouse #1, which are given along the white dashed lines that are crossing the center of the tumor in the coronal image.

**Figure 4:**
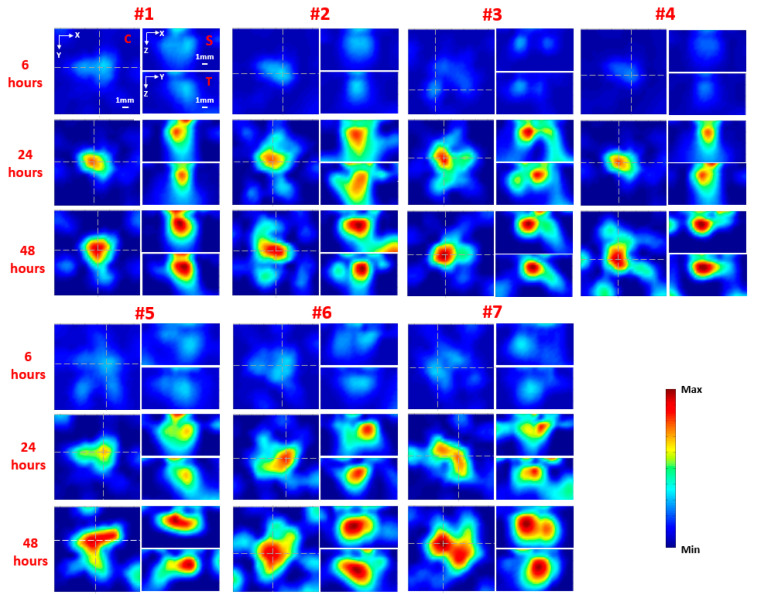
Reconstructed FMT images along representative coronal (C), sagittal (S), and transverse (T) planes for the seven mice in the targeted (NIR 830-ATF-IONP) group at 6, 24, and 48 h post injection of nanoprobes. The C slice is indicated in the first image for mouse #1 (upper left), while the S and T slices are also noted in the other two images in the first set of images for mouse #1, which are given along the white dashed lines that are crossing the center of the tumor in the coronal image.

The planar fluorescence images of each mouse at 6, 24, and 48 h following injection are shown in Figure 1 for the nontargeted group (nontargeted NIR 830-IONP) and Figure 2 for the targeted group (NIR 830-ATF-IONP), respectively. Correspondingly, the recovered FMT images of each mouse at the three different time points are shown in Figure 3 for the nontargeted group and Figure 4 for the targeted group, respectively. For comparison between the planar fluorescence images, the exposure time of the Charge-Coupled Device (CCD) camera was constantly set to 0.4 s. Contrary to the planar fluorescence images, the FMT images shown in Figure 3 and Figure 4 provide a three-dimensional (3D) distribution of the fluorophores in the tissues, including the coronal, transverse, and sagittal planes crossing the center of the tumors. The results from both imaging modalities show that a high level of florescence signal intensity was found in the tumors that received targeted NIR 830-ATF-IONP and suggest effective accumulation of the nanoprobes in the tumors at the three time points. As expected, tumors that received an injection of NIR 830-ATF-IONP had the strongest fluorescence signal, compared with the tumors of the mice that received an injection of nontargeted NIR 830 nanoparticles after 24 and 48 h. We noticed that the FMT results were highly consistent with the results of planar fluorescence imaging. Moreover, the FMT images provided depth information on the tumors that was not available from planar fluorescence imaging.

**Figure 5:**
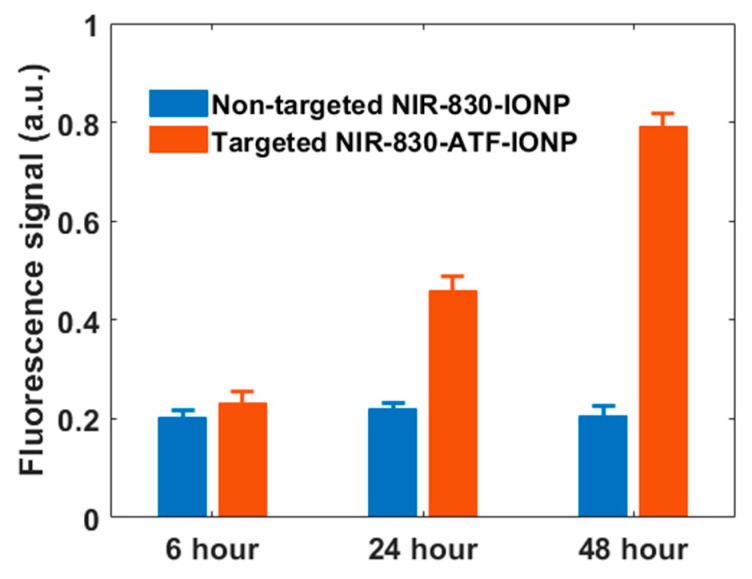
Quantitative plot of FMT recovered mean fluorescence signals from the two animal groups at 6, 24, and 48 h following the injection of nontargeted NIR 830-IONP (blue) and targeted NIR 830-ATF-IONP (orange).

Figure 5 shows the mean fluorescence signal enhancement in the tumor from the nontargeted and targeted animal groups as a function of time after injection of nontargeted and targeted nanoprobes, respectively. We observed weak fluorescence signals in the tumor masses 6 h after administration of the nanoprobes in both groups, with slightly higher signals when using targeted nanoprobes. However, compared with the nontargeted group, the signal intensity for the targeted group increased about 2.5-fold at 24 h and 4-fold at 48 h after the injection. In contrast, we did not see a significant change in fluorescence signals for the nontargeted group at the three time points. The results show the specific accumulation of targeted nanoprobes in the tumor tissues with high expression of uPAR, which reached the peak at approximately 48 h post injection and dropped off later slowly [21]. The weak fluorescence signal from the tumors with nontargeted probes shows the accumulation of a small amount of nanoprobes in the tumor tissue, and the decrease of the signal after 24 h suggests the slow removal of nontargeted nanoprobes from the circulatory system. Thus, approximately 48 h post-injection of NIR 830-ATF-IONP should be the best window for imaging.

## 3. Discussion

Breast cancer is highly heterogeneous at the cellular and molecular levels [23]. Previous studies show that uPAR, a cellular receptor, is expressed in 60%–90% of invasive breast cancer tumors; 54% of early stage ductal carcinoma in situ (DCIS) cells express a high level of uPAR [24,25]. Interaction of urokinase plasminogen activator (uPA) with uPAR leads to the internalization of the ligand–receptor complex, suggesting an advantage of enhancing tumor accumulation of the nanoparticles [21,26]. Furthermore, uPAR is highly expressed in several tumor-associated stromal cell types, including active macrophages, angiogenic endothelial cells, and active fibroblasts [24]. Since tumor margins are enriched in active tumor stromal cells, targeting uPAR will lead to the accumulation of imaging probes in the tumor edge, which facilitates detection of tumor margins by optical imaging [27]. As we expected, the results presented above demonstrate that NIR 830-ATF-IONP is a highly specific optical imaging probe with high sensitivity and specificity that can be used for targeted molecular imaging. In addition, it is important to point out the fact that NIR 830-ATF-IONP is a multifunctional imaging probe providing not only the fluorescence signal from NIR 830 dye but also a strong absorption of light due to the IONPs. This unique character means it can also be used as a contrast agent for other imaging modalities, such as photoacoustic imaging (PAI) and magnetic resonance imaging (MRI) [28]. Therefore, we are working on a novel dual-modality imaging endoscopic device that integrates FMT and PAI. We expect to obtain both FMT and PAI images from a single small endoscope in the near future.

Mammography, an X-ray examination of the breast, is the most common screening method for breast cancer, although it still has difficulties in detecting breast tumors in women with dense breast tissue [29]. Breast magnetic resonance imaging (MRI) is also used as a diagnostic technology for women who have a high risk of breast cancer and for women who are not willing to be exposed to radiation [30]. However, neither technology can be used as an intraoperative instrument in surgery due to their bulky size, expensive cost, and poor temporal resolution [31,32]. Ultrasound is usually used as a complement to mammography in the diagnosis of breast cancer. While it has good temporal resolution and a lower cost, the poor sensitivity and specificity make it unsuitable to be used as an intraoperative device for breast cancer detection [33]. With its good tissue penetration and high spatial resolution, photoacoustic imaging (PAI) is emerging as a promising optical imaging technology in the detection of breast cancer. Compared with PAI, FMT has better sensitivity and specificity due to the usage of targeted NIR dyes. The results of our previous study showed that FMT can easily detect strong fluorescent signal from 2 µM indocyanine green (ICG) [34] or 0.02 pM NIR 830-IONPs [28], while there was no detectable photoacoustic (PA) signal from 2 µM ICG or 0.1 pM NIR 830-IONPs. In the last few decades, significant progress has been made in the development of miniaturized FMT devices. He et al. [35] reported a MEMS-based FMT probe with a diameter of 25 mm. A handheld FMT device with a diameter of 16 mm using many optical fiber bundles was presented in [36]. In Yang et al. [13], a miniaturized FMT device called an FMT pen was reported: In this study, the highly miniaturized size of this novel FMT endoscope made it easy to be inserted into the body cavity during surgery for tumor removal. This capability can effectively assist surgeons to find those small residual tumors that are not easily reached by conventional imaging techniques. While it is promising, we noticed that the data acquisition time needed for FMT is relatively long, which may be a concern for its clinical applications. There are two possible solutions to speed up the data acquisition process. First, a CCD camera with higher sensitivity can speed up the data acquisition process by shortening the exposure time. Second, a fiberscope with a larger diameter and more optical fibers placed inside can acquire more fluorescence signals from the target and thus further shorten the exposure time of a CCD camera. Finally, because more detection data could be extracted from a fiberscope, it is possible to shorten the data acquisition time using fewer source positions. Combined with an optimized reconstruction algorithm, it is expected that the imaging process could be speeded up dramatically.

## 4. Materials and Methods

### 4.1. Miniaturized FMT Endoscope

**Figure 6:**
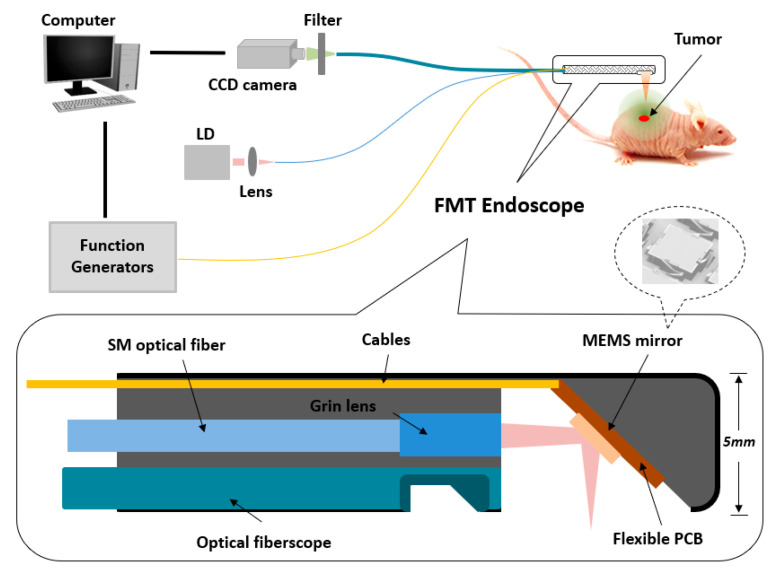
Schematic of the miniaturized FMT endoscopic system.

Figure 6 shows a schematic of the miniaturized FMT endoscopic system with the insert showing the endoscope with a diameter of 5 mm mainly consisting of a MEMS scanning mirror and an optical fiberscope. A laser beam from a CW laser diode (780 nm, Thorlabs) was first coupled to a single-model optical fiber and then focused by a micro grin lens. Through a homemade MEMS mirror (aperture size: 1 × 1 mm^2^ and footprint size: 2 × 2 mm^2^) controlled by two function generators (Tektronix, Afg3022B, OR), the focused laser beam was reflected and redirected to different positions on the surface of animal skin precisely by applying different voltages to the four actuators made from a lateral-shift-free large-vertical-displacement (LSF-LVD) design [37,38]. Finally, the fluorescence light from the tissue was collected by an optical fiberscope (Myriad Fiber Imaging Tech, MA, 1.2 mm diameter with 30 K optical fibers) and then captured by a highly sensitive electron-multiplying CCD (EMCCD) camera (Andor, IXON) coupled with an 830 nm band-pass filter (Edmund Optics, NJ). In this study, the total imaging domain was 1 × 1 cm^2^, including 25 detection and 12 source positions, respectively. A Labview program was used to control and synchronize the whole system including the function generator and the EMCCD camera. The total data acquisition time for each image was variable and depended on the intensity of fluorescence light from the target, which was from 9 s to 12 s in this study. The 3D fluorescence images were reconstructed using a finite-element-based algorithm described in detail previously [39].

### 4.2. Nanoparticle

**Figure 7:**
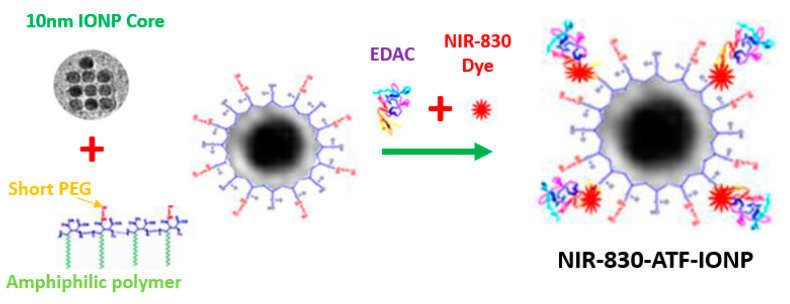
Schematic of the production of receptor targeted imaging nanoparticles.

Magnetic iron oxide nanoparticles (IONP) were prepared by Ocean Nanotech LLC using iron oxide powder as the iron precursor, oleic acid as the ligand, and octadecene as the solvent [40]. The IONP particles, with a core size of 10 nm, were coated with amphiphilic polymers using a similar method as reported previously [41]. Amino-terminal fragment (ATF) peptides were conjugated to the surface of IONP mediated by the cross-linking of carboxyl groups on the coated polymer to amino side groups on the ATF peptides, as shown in Figure 7. Briefly, polymer-coated IONPs were activated by ethyl-3-dimethyl amino propyl carbodiimide (EDAC, Pierce, Rockford, IL, USA) and sulfo-NHS for 15 min. After purification using Nanosep 100k OMEGA (Pall Corp, Ann Arbor, MI, USA), NIR 830 dye-conjugated (absorption peak: 800 nm; emission peak: 830 nm) ATFs were added in a molar ratio of IONP:ATF of 1:20 in pH 7.0 PBS at 4 °C overnight, generating NIR 830-ATF-IONP. The final NIR 830-ATF-IONP conjugates were purified using Nanosep 100 k column filtration. The hydrodynamic size of NIR 830 ATF-IONP was approximately 18 nm, which was determined by dynamic light scattering (Zetasizer, Malvern Panalytical, Westborough, MA, USA).

### 4.3. Animal Model

A human TNBC PDX model derived from one TNBC patient was used in this study. The early passage PDX tumors from a TNBC patient’s tissue sample had been established in Dr. Yang’s lab. Early passage tumor fragments (<5 passages) were frozen in liquid nitrogen and were re-implanted subcutaneously (s.c.) to generate PDX tumors in nude mice. Over 85% of nude mice had s.c. tumor growth within 3 weeks following implantation. All procedures described above were approved by the University of South Florida Animal Care and Use Committee and conducted in accordance with the National Institutes of Health Guide for the Care and Use of Experimental Animals.

## 5. Conclusions

In summary, we developed a novel FMT endoscopic device utilizing a MEMS mirror to perform 2D scanning and an optical fiberscope to acquire fluorescence signals, and we used uPAR targeted nanoprobes as a contrast agent. Twelve nude mice bearing PDX tumors were divided into targeted and nontargeted groups and were imaged at 6, 24, and 48 h following the injection of nanoprobes. The results show that the FMT recovered fluorescence signal with targeted nanoprobes increased approximately 2.5-fold at 24 h and 4-fold at 48 h after injection of nanoprobes when compared with nontargeted nanoprobes. The findings from this study suggest that the combination of our novel FMT endoscope with the highly sensitive and specific uPAR targeted nanoprobes offers great potential for helping surgeons find and remove residual tumor lesions.

## Abbreviations

FMT	Fluorescence Molecular Tomography
MRI	Magnetic Resonance Imaging
NIR	Near Infrared
IONP	Iron-Oxide Nanoparticle
TNBC	Triple-Negative Breast Cancer
PDX	Patient-Tissue-Derived Xenograft
uPAR	urokinase Plasminogen Activator Receptor
ATF	Amino-Terminal Fragment
CCD	Charge-Coupled Device
EMCCD	Electron-multiplying CCD
MEMS	Microelectromechanical Systems
